# Comparison of Machine Learning Algorithms for Heartbeat Detection Based on Accelerometric Signals Produced by a Smart Bed

**DOI:** 10.3390/s24061900

**Published:** 2024-03-15

**Authors:** Minh Long Hoang, Guido Matrella, Paolo Ciampolini

**Affiliations:** Department of Engineering and Architecture, University of Parma, 43124 Parma, Italy; paolo.ciampolini@unipr.it

**Keywords:** heartbeat detection, machine learning, deep learning, artificial intelligence algorithm, accelerometer sensor, smart bed

## Abstract

This work aims to compare the performance of Machine Learning (ML) and Deep Learning (DL) algorithms in detecting users’ heartbeats on a smart bed. Targeting non-intrusive, continuous heart monitoring during sleep time, the smart bed is equipped with a 3D solid-state accelerometer. Acceleration signals are processed through an STM 32-bit microcontroller board and transmitted to a PC for recording. A photoplethysmographic sensor is simultaneously checked for ground truth reference. A dataset has been built, by acquiring measures in a real-world set-up: 10 participants were involved, resulting in 120 min of acceleration traces which were utilized to train and evaluate various Artificial Intelligence (AI) algorithms. The experimental analysis utilizes K-fold cross-validation to ensure robust model testing across different subsets of the dataset. Various ML and DL algorithms are compared, each being trained and tested using the collected data. The Random Forest algorithm exhibited the highest accuracy among all compared models. While it requires longer training time compared to some ML models such as Naïve Bayes, Linear Discrimination Analysis, and K-Nearest Neighbour Classification, it keeps substantially faster than Support Vector Machine and Deep Learning models. The Random Forest model demonstrated robust performance metrics, including recall, precision, F1-scores, macro average, weighted average, and overall accuracy well above 90%. The study highlights the better performance of the Random Forest algorithm for the specific use case, achieving superior accuracy and performance metrics in detecting user heartbeats in comparison to other ML and DL models tested. The drawback of longer training times is not too relevant in the long-term monitoring target scenario, so the Random Forest model stands out as a viable solution for real-time ballistocardiographic heartbeat detection, showcasing potential for healthcare and wellness monitoring applications.

## 1. Introduction

Nowadays, technologies dedicated to monitoring people’s physiological parameters and behaviors have become strategic in the field of Digital Prevention (DP), especially with reference to chronic disease prevention or early detection. The development of DP techniques and methods is the aim of the Digital Lifelong Prevention (DARE) project [1,2], in which the activities illustrated in this article are framed.

Cardiovascular diseases [3,4] are among the pathologies that can most benefit from the use of DP techniques [5,6,7]. In such cases, the importance of continuous health monitoring has led to the development of innovative methods for accurate heart rate detection [8,9,10] that is considered a reliable indicator for the assessment of cardiac functions [11].

For these reasons, many wearable devices were developed to track heart pulse behavior. In article [12], the gyroscope and accelerometer are utilized with the Arduino microcontroller, strapped to the torso, and transmitted sensor signals for heart rate measurements. Smartwatches are the most common heart rate monitoring tools [13,14,15,16]. They use photoplethysmography (PPG), a non-invasive optical technique to measure blood volume changes in the microvascular bed of tissue. Nevertheless, there are some known issues related to these device utilizations, especially for monitoring elderly users: smartwatches need to be recharged frequently, it can be annoying to keep them in contact with the body skin, or the user can simply forget to wear it. In addition, electrocardiography (ECG) is a common method for detecting the heart’s electrical activity during its contraction and relaxation phases. ECG is typically done by placing electrodes on the skin to capture the signals. However, this technique is quite invasive: it requires a long preparation procedure, a specific ECG machine, ECG electrodes, conductive gel, and skin-prepping solution.

Thus, the approach in our paper is based on the specific development of the smart bed system, which provides a non-invasive and uninterrupted monitoring solution while the individual is asleep. The smart bed system utilizes a 3-axis MEMS accelerometer [17] to collect acceleration signals, which generates a comprehensive dataset for training and testing Artificial Intelligence (AI) models.

On the other hand, Machine learning [ML] [18,19,20] approaches have demonstrated their high potential effectiveness in healthcare monitoring [21]. In [22], a support vector machine (SVM) model was implemented to predict the mental stress condition from the obtained heart rate. Another study [23] also used SVM to determine whether someone has a risk of heart disease based on the retrieved heart rate. In addition, ML techniques have considerably contributed to detecting, predicting, or monitoring cardiovascular disease [24]. The paper [25] proposes a heartbeat classifier trained by ML for continuous heartbeat monitoring using the Polar H10, a chest strap type. However, all these wearable devices still need to be equipped with the users for work. Approaches based on the joint use of AI algorithms and ballistocardiographic signals have already been investigated in previous works [26,27,28].

Therefore, our research develops a smart bed system to achieve reliable, comfortable heart rate monitoring for a long time with no burdens for the users: they just need to lie on the bed without any physical contact with sensors or need to recharge to have their heart rate monitored.

In [29], the research utilizes resistive pressure sensors under the mattress for heartbeat tracking. These sensors can measure slight pressure changes during breathing and heartbeat, which can affect the person’s positioning on the mattress. Another research [30] tracks heart rate during sleep using the “Out of Center Sleep Testing” (OCST) system, including force-sensitive resistors. The ballistocardiogram signal is processed using the discrete wavelet transform and the Butterworth bandpass filter to measure the heart rate.

In such papers, however, results are mostly reported in the frequency domain (i.e., beats per minute), and no discussion about the validation of single beats is given. Accurate time positioning of detected peaks is relevant to the evaluation of heart rate variability and in detecting some arrhythmias; hence, we apply the ML algorithms to accomplish precise heartbeat time tracking and evaluate system accuracy on such a basis. This research explores the utilization of both conventional ML and sophisticated Deep Learning (DL—a subset of machine learning methods) [31,32,33,34] to identify heart pulses.

Concerning mentioned papers [29,30], it is also worth mentioning that the adoption of either piezoresistive or force-sensitive sensors necessarily relies on the proper positioning of sensing devices within the bed structure, also depending on the user’s body features and posture and possibly requiring some caution when making up or sanitizing the bed By using a solid-state accelerometer, instead, we are much less dependent on sensor placement, and more practical and sustainable placements are made possible.

To guarantee the strength and capacity to apply to various situations of the models, K-fold cross-validation is utilized. This methodology guarantees that the models are subjected to testing on several subsets of the data, enabling a thorough assessment of their performance. The evaluation determines the most efficient method for detecting cardiac pulses. The encompassed techniques are Logistic Regression (LR) [35,36], Linear Discriminant Analysis (LDA) [37,38], K-Nearest Neighbour Classification (KNN) [39,40], Classification and Regression Trees (CART) [41,42], Naive Bayes (NB) [43,44], Support Vector Machines (SVMs) [45,46], and Random Forest (RF) [47,48] and Deep Neural Network [49,50]. Every algorithm possesses distinct strengths and qualities that are relevant to the specific job. The paper aims to allow for a comparison of their merits and appropriateness for heart rate detection in a smart bed system, in terms of performance and time operation.

The paper is organized as follows: the 1st part is about setup and devices, demonstrating the data acquisition process. In the next part, the main working principle of each algorithm will be described, together with the evaluation metrics. The last part is about the result, analysis, and conclusion.

## 2. Materials and Methods

### 2.1. Setup and Devices

**Figure 1:**
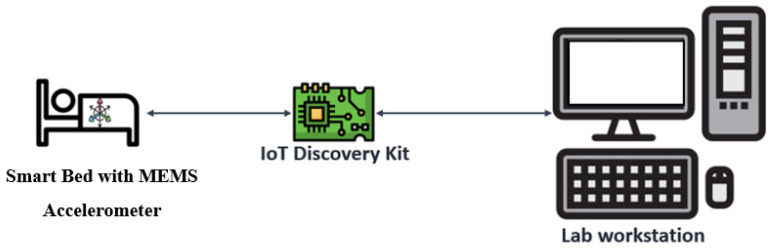
Data acquisition diagram.

To make it “smart”, a standard bed was equipped with a 3-axis MEMS Accelerometer with 20-bit acceleration resolution [51], as shown in Figure 1. Acceleration data has been acquired by an IoT kit STM32 B-L475-IOT01A microcontroller board [52] via serial peripheral interface communication (SPI) at the sample rate of 250 Sa/s. Using a ballistographic approach, it is possible to analyze the acceleration data signals to detect the heartbeat. During the acquisition campaign, the MCU board was connected evenly to a finger pulse sensor [53] to provide a ground truth for ML models. Finally, all the accelerometers’ tracks were transferred in real-time to a workstation to be recorded in text files. Afterwards, such files have been used to train and test the ML algorithms developed by Python language [54], taking advantage of its specific AI libraries: Scikit-learn [55], Keras from TensorFlow platform [56].

**Figure 2:**
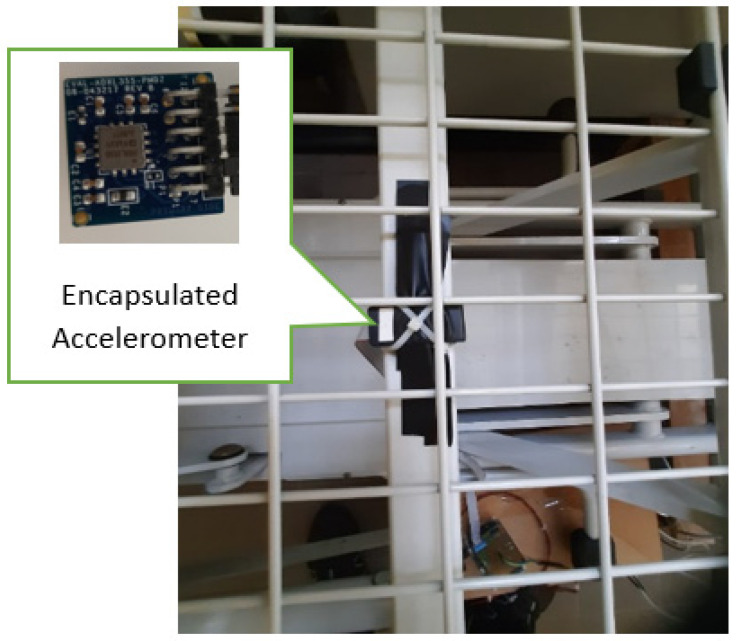
Encapsulated accelerometer under the bed frame and its orientation.

**Figure 3:**
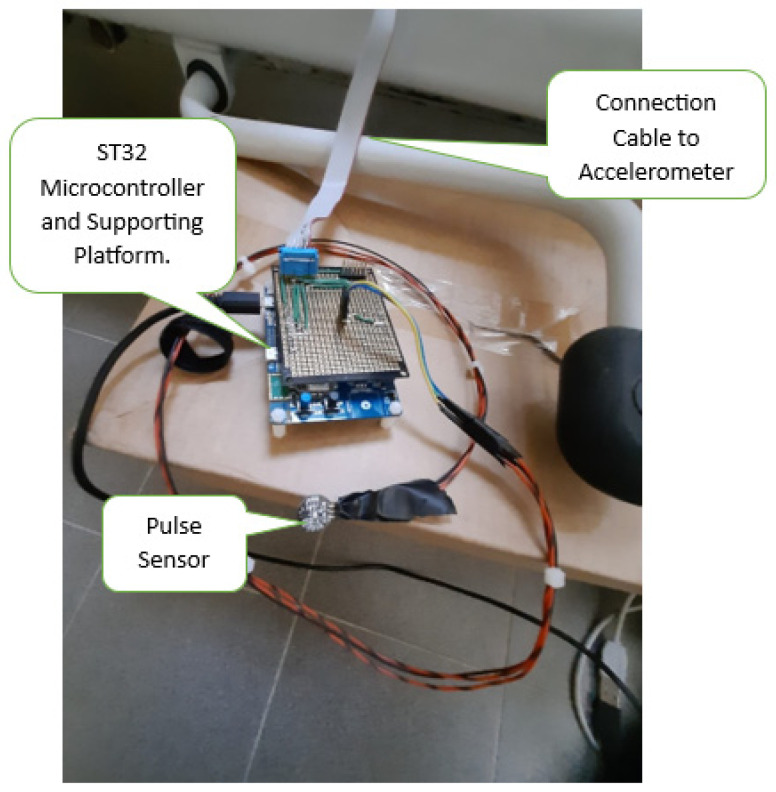
STM32 Microcontroller platform for sensor pulse and accelerometer.

The micro-electromechanical system (MEMS) accelerometer [57,58,59] is encapsulated to be appropriately mounted under the bed frame as shown in Figure 2, and it is connected to the microcontroller through an SPI connection. As demonstrated in Figure 3, the ADXL355 sensor provides acceleration data in a 32-bit digital format from X, Y, Z axes. The sample rate was configured at 250 Sa/s. At the same rate, finger pulse sensor data are acquired by the 12-bit A0 channel of the ADC (Analog to Digital Converter) present inside the MCU. All the acquired data (X, Y, Z, and finger pulse) are transmitted to the workstation by a serial connection at the baud rate of 921,600.

**Figure 4:**
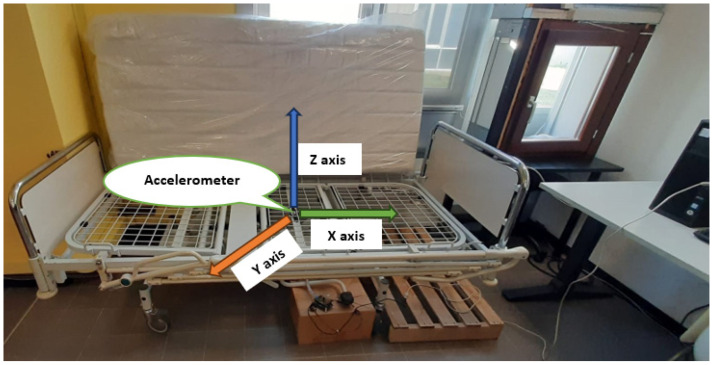
Smart bed under testing.

All the devices were installed under the bed as shown in Figure 4.

The finger pulse sensor operates by emitting a green light (about 550 nm) onto the finger and quantifying the level of light reflected using a photosensor. The property of arterial blood’s oxygen-hemoglobin is to absorb green light. With each heartbeat, the finger pumps blood, causing a change in the amount of reflected light. This results in a fluctuating waveform at the output of the photosensor. The signal undergoes filtration using an R/C filter, followed by an operational amplifier amplification to generate the output signal.

**Figure 5:**
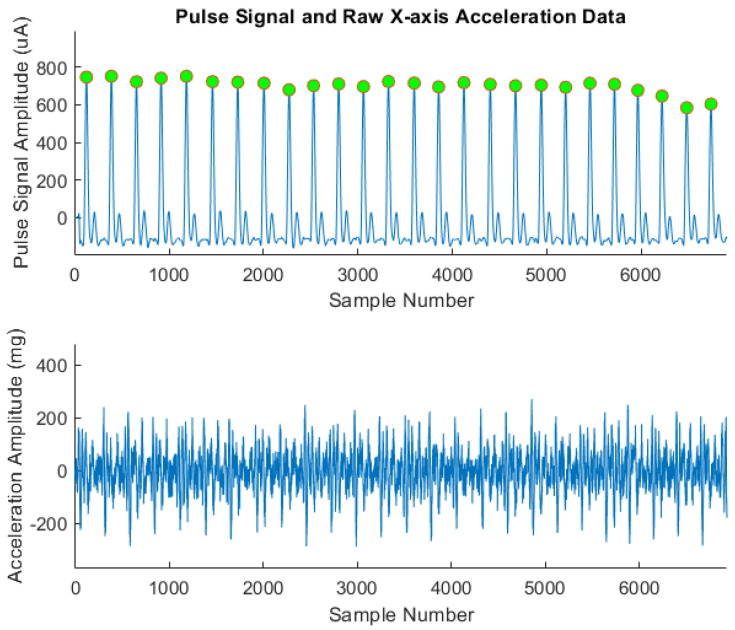
Pulse signal and original *X*-axis acceleration data on right side position.

The information from the pulse sensor will be processed to infer when the “heartbeat” event occurred. This information is necessary to provide the “ground truth” in the training of ML algorithms because it is more explicit, less noisy, and more precise with respect to the original acceleration data, as shown in Figure 5. Each peak is highlighted by a green dot, corresponding to a heartbeat. 

### 2.2. Data Processing and ML Features

A specific dataset was produced to train and test the algorithms: 10 people participated in a laboratory data acquisition campaign and 2 h of ballistographic signals were recorded to create reliable and resilient heartbeat recognition models. Each person’s measurements were acquired in 4 lying positions: 1. prone, 2. back, 3. right side, and 4. left side. Since the research aims to monitor people during sleep, only resting conditions were tested, with no particularly intense physical activity involved before or during the test. They did not do any fast walking or running, they stayed at rest or only walk normally at least 15 min before the tests. For each position, accelerations were acquired for 180 s. The acceleration acquisition rate has been set at 250 Sa/s (generally, the human heart rate is between 40 and 120 beats per minute, equivalent to 0.6–2 Hz [60].

Hence, each participant has produced 4 files (one for position) with 45,000 lines (180 s per 250 Sa/s) and 4 columns (*X*-axis, *Y*-axis, and *Z*-axis and finger pulse) for an overall 40 files of raw data. In the next phase, raw data were elaborated before being used to train ML algorithms. A bandpass filter digitally filters raw acceleration along *X*-axis, *Y*-axis, *Z*-axis, and finger pulse data at [0.5–20] Hz to remove unnecessary noise.

After the filter, Δacc is defined as the absolute value of the difference between 2 consecutive acceleration samples. All the signal values Δacc were converted into absolute values before generating the ML features. A window of 125 samples is exploited to extract the relevant features listed below. ML model will predict the presence or absence of heartbeat after each window of 125 samples. Therefore, there are 14,400 windows for all signals, calculated as follows:

(1) $$\begin{array}{l} N_{\mathrm{Window}}=\frac{\mathrm{total}\;\mathrm{sample}}{\mathrm{sample}\;\mathrm{per}\;\mathrm{window}}\\ =\frac{\mathrm{sample}\;\mathrm{rate}\times \mathrm{acquisition}\;\mathrm{time}\times \mathrm{position}\;\mathrm{number}\times \mathrm{participant}\;\mathrm{number}\;}{\mathrm{sample}\;\mathrm{per}\;\mathrm{window}}\\ =\frac{250\times 180\times 4\times 10}{125}=14400 \end{array}$$

where: $N_{\mathrm{Window}}$ is the window number.

The list of Input features includes:

- Xsum, Ysum, Zsum are the sum of acceleration along *X*, *Y*, and *Z*-axes for each window.
- Xstd, Ystd, Zstd are the standard deviation of acceleration along *X*, *Y*, and *Z*-axes for each window.
- Xmax, Ymax, Zmax are the maximum value of acceleration along *X*, *Y*, *Z*-axes for each window.

Since heartbeat has the most substantial impact on Xacc, so 3 other features are calculated based on the difference between 2 consecutive accelerations (Δacc) are calculated as follows:

- ΔXsum is the sum of Δacc along *X*-axis for each window.
- ΔXstd is the standard deviation of Δacc along *X*-axis for each window.
- ΔXmax is the maximum value of Δacc along *X*-axis for each window.

The Output consists of Heartbeat detection per each window, according to the following encoding (see also Table 1):

- 0: No heartbeat detected
- 1: Heartbeat detected

### 2.3. ML Algorithms

There are seven powerful algorithms in consideration for ML classification: logistic regression (LR), linear discriminant analysis (LDA), K-nearest neighbor classification (KNN), classification and regression trees (CART), Naive Bayes (NB), support vector machines (SVMs), and Random Forest (RF).

#### 2.3.1. Logistic Regression

**Figure 6:**
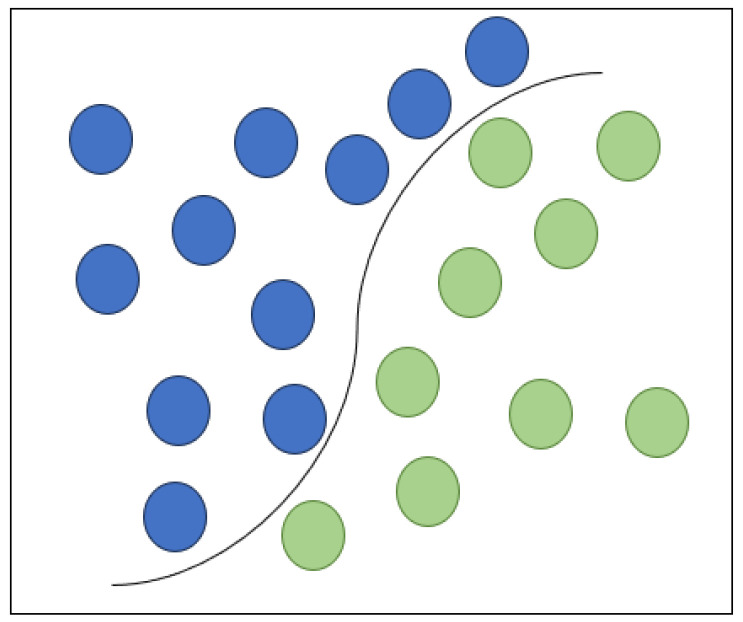
Logistic regression illustration in binary classification.

Logistic regression is a statistical method used for binary classification, where the goal is to predict the probability that an instance belongs to a particular class. Despite its name, logistic regression is a classification algorithm rather than a regression algorithm. It’s widely used for problems where the dependent variable is binary, meaning it has two possible outcomes, as illustrated in Figure 6.

In logistic regression, the relationship between the features and the positive class probability is modeled using the logistic function (also called the sigmoid function). The logistic function is an S-shaped curve that maps any real-valued number to the range [0, 1]. The formula for the logistic function is:

(2) $$P\left(Y=1\right)=\frac{1}{1+e^{-\left(\beta_{0}+\beta_{1\;}X_{1}+\cdots \beta_{n\;}X_{n\;}\right)}}$$

where:

- P(Y = 1) is the probability that the dependent variable Y is equal to 1 (positive class).
- e is the base of the natural logarithm.
- β_0_, β_1_, …, β_n_ are the coefficients to be learned from the training data.
- X_1_, …, X_n_ are the input features.

The goal during training is to find the values of β_0_, β_1_, …, β_n_ that maximize the likelihood of the observed data using optimization algorithms like gradient descent.

Once the model is trained, the probability P(Y *=* 1) is calculated for each instance, and a decision rule is applied to classify the instance into one of the two classes based on a chosen threshold (commonly 0.5). For example, if P(Y = 1) is greater than or equal to 0.5, the instance is classified as the positive class; otherwise, it’s classified as the negative class.

#### 2.3.2. Linear Discrimination Analysis

**Figure 7:**
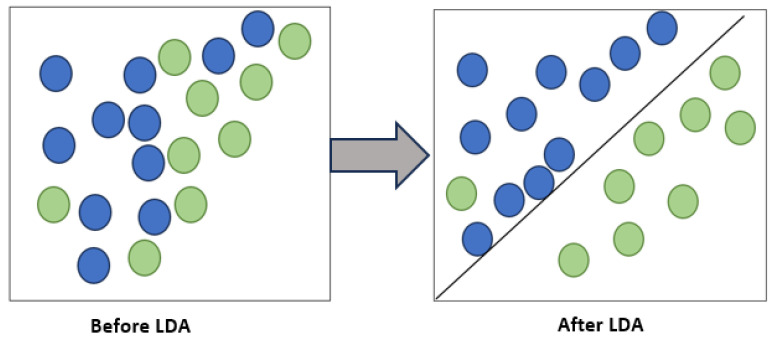
LDA illustration.

The primary goal of LDA is to maximize the separation between the means of different classes while minimizing the variance within each class as shown in Figure 7. It achieves this by projecting the data onto a lower-dimensional subspace.

Firstly, the model calculates the mean vectors for each class, representing the average feature values for each class. The next stage is scattering matrix computation. Calculate the within-class scatter matrix (S_W_) and the between-class scatter matrix (S_B_). S_W_ measures the spread of data within each class. S_B_ measures the spread between class means.

(3) $$S_{W}=\overset{c}{\underset{i=1}{\sum }}\overset{n_{i}}{\underset{j=1}{\sum }}\left(x_{\mathrm{ij}}-{\;\mu }_{\mathrm{ij}}\right){\left(x_{\mathrm{ij}}-{\;\mu }_{\mathrm{ij}}\right)}^{T}$$

(4) $$S_{B}=\overset{c}{\underset{i1}{\sum }}N_{i}\left(\mu_{\mathrm{ij}}-\;\mu \right){\left(x_{\mathrm{ij}}-{\;\mu }_{\mathrm{ij}}\right)}^{T}$$

where:

- c is the number of classes.
- N_i_ is the number of instances in class i.
- x_ij_ is the j-th instance of class i.
- μ_i_ is the mean vector of class i.
- μ is the overall mean vector.

The next step is Eigenvectors and Eigenvalues computation, solving the generalized eigenvalue problem to find the eigenvectors (*v*) and corresponding eigenvalues (*λ*) of $S_{W}^{-1}$ S_B_. Then, the model sorts out the eigenvectors in descending order based on their corresponding eigenvalues. These eigenvectors form the new axes of the subspace. The subsequent stage projects the original data onto the subspace formed by the top *k* eigenvectors, where *k* is the desired dimensionality (usually *k* = 1 for binary classification).

For binary classification, a common decision rule involves thresholding the projected values.

- If ≥ 0.5, classify as Class 1.
- If < 0.5, classify as Class 2.

#### 2.3.3. K Nearest Neighbours

**Figure 8:**
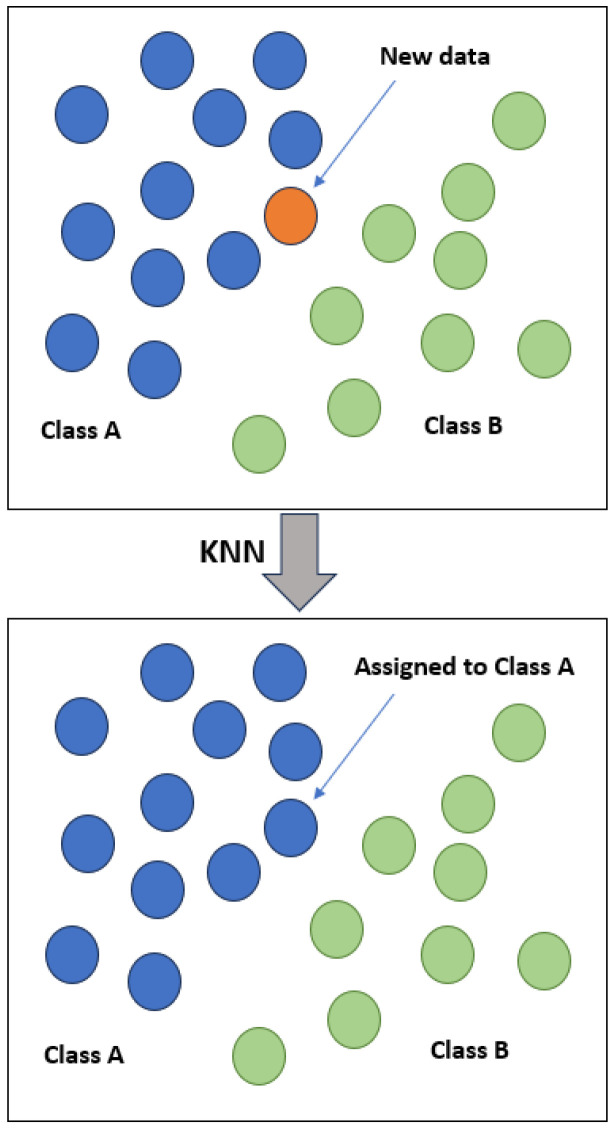
KNN illustration.

KNN model predicts the class of a new data point based on the majority class of its k nearest neighbours in the feature space, as shown in Figure 8. In the first state, KNN stores the training dataset. New data points are given to classify; the algorithm calculates its distance to all other points in the training dataset using a distance metric (commonly Euclidean distance). The algorithm identifies the k training instances with the shortest distances to the new data point. For classification, the algorithm counts the number of instances in each class among the K neighbours, depending on majority voting. As a result, the new data point is assigned the most common class among its k nearest neighbours.

Here, K is the number of neighbours:

- The choice of K is a hyperparameter that needs to be specified. It determines how many neighbours influence the classification decision.
- A smaller K (e.g., 1 or 3) makes the algorithm more sensitive to noise but can capture local patterns.
- A larger K (e.g., 10 or 20) provides a smoother decision boundary but may miss local variations.

Decision Rule:

- For binary classification, the decision rule involves a majority vote among the k nearest neighbors.
- If k is odd, there will be a clear majority.
- If k is even, a tie-breaking rule may be needed.

#### 2.3.4. Classification and Regression Trees

**Figure 9:**
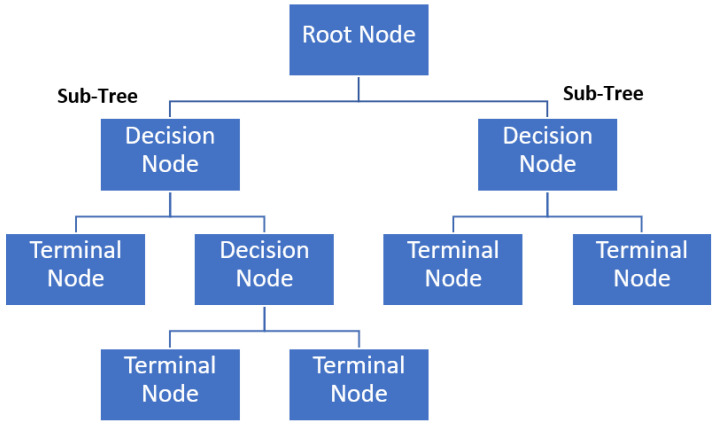
CART illustration.

A decision tree is a hierarchical structure consisting of nodes, where each node represents a decision or a test on a particular feature. The tree structure is built recursively based on the data. At each node, the algorithm selects the feature and a threshold to split the data into two subsets. The goal is to make the subsets as pure as possible regarding the target variable (class labels). The algorithm searches for the best split by evaluating different features and thresholds. Standard impurity measures for binary classification include Gini impurity and cross-entropy [61]. The splitting process continues until a stopping criterion is met, such as a maximum depth, a minimum number of samples in a leaf, or reaching a pure node (all instances in a node belong to the same class). To make predictions for new instances, they traverse the tree from the root to a leaf node based on the feature values. The predicted class is often determined by the majority class in the leaf node, as shown in Figure 9.

#### 2.3.5. Naive Bayes

Naive Bayes is a probabilistic machine learning algorithm that is commonly used for binary classification tasks. The NB algorithm works based on Bayes’s theorem and makes the naive assumption that the features are conditionally independent given the class. Bayes’ theorem relates the conditional and marginal probabilities of random events. For binary classification, it can be expressed as follows:

(5) $$P(Y|X)=\frac{P\left(X|Y\right)P\left(Y\right)\;}{P\left(X\right)}$$

where:

- Y is the class variable (e.g., 0 or 1).
- X is the vector of feature variables.

Naive Bayes assumes that the features are conditionally independent given the class that allows for a computationally efficient and interpretable model.

**Figure 10:**
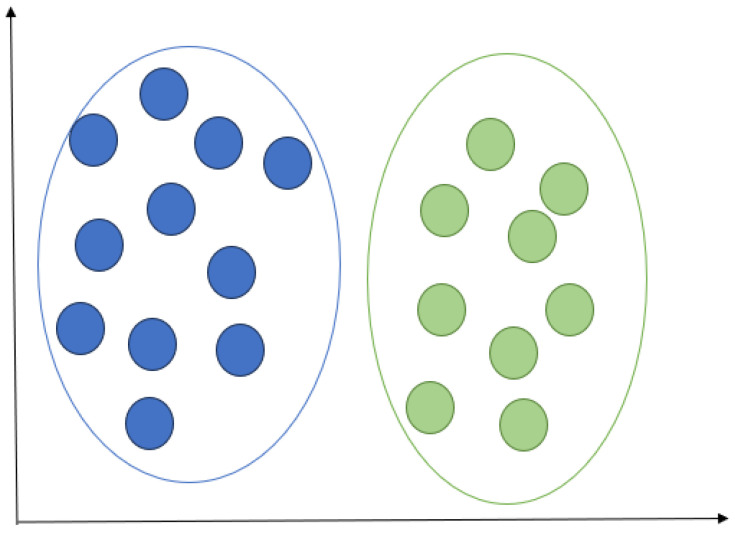
Naive Bayes illustration.

The model estimates the probabilities P(X∣Y) and P(Y) from the training data in the training process. In prediction, the probability of each class Y will be calculated for the new instances with features X calculated. Finally, the NB model assigns the instance to the class with the highest probability, as demonstrated in Figure 10.

#### 2.3.6. Support Vector Machines

**Figure 11:**
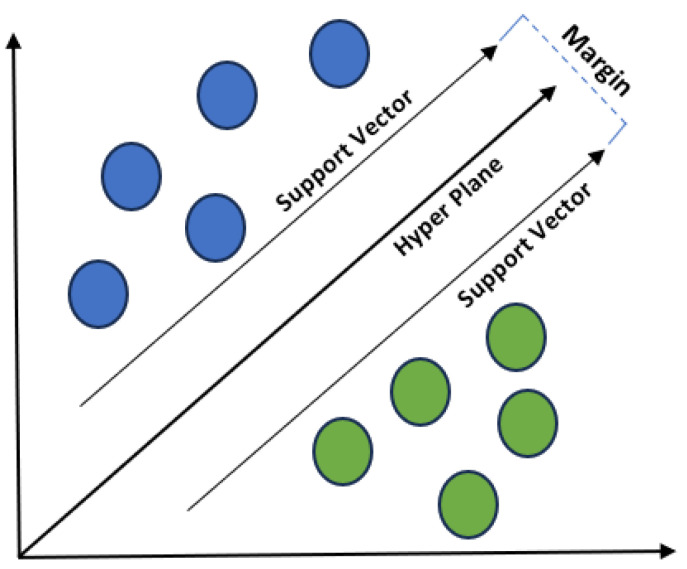
SVM illustration.

SVM aims to find a hyperplane that best separates the data into two classes. The hyperplane is chosen to maximize the margin, which is the distance between the hyperplane and the nearest data points from each class. SVM seeks to find a hyperplane that separates the data into two classes. If the data is linearly separable, SVM looks for the hyperplane with the maximum margin. The margin is the distance between the hyperplane and the nearest data point from each class. SVM aims to maximize this margin. Support vectors are the data points that are closest to the hyperplane and are crucial in determining the optimal hyperplane. See Figure 11.

#### 2.3.7. Random Forest

RF is an ensemble learning algorithm that is effective for both classification and regression tasks. This algorithm works by constructing a multitude of decision trees during training and outputs the class, that is the mode of the classes (classification) or the mean prediction (regression) of the individual trees. RF introduces randomness both in the selection of data samples and the features used for decision tree construction, which often leads to improved generalization performance.

**Figure 12:**
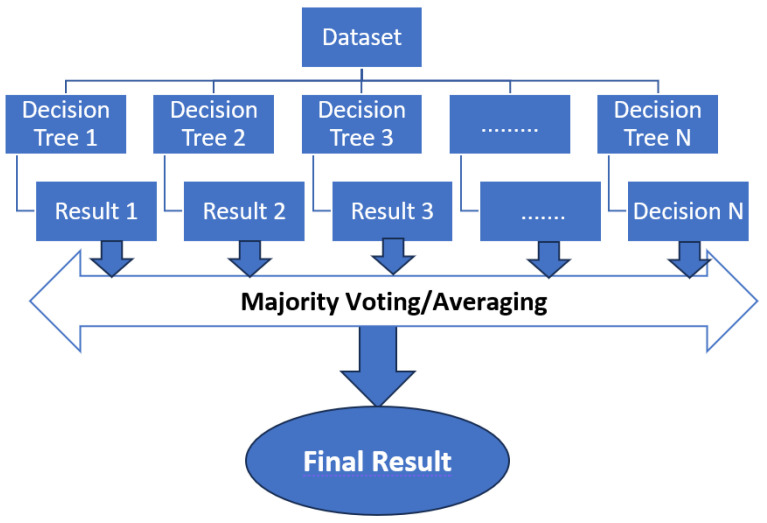
Random Forest illustration.

RF builds each tree on a random subset of the training data by sampling with replacement as bootstrapped sampling. At each decision tree node, a random subset of features is considered for the split to help de-correlate the trees and prevent overfitting. Each tree in the forest is grown deep and is unpruned, resulting in low bias but high variance. The ensemble of trees works together to reduce the overall variance. For classification tasks, the final prediction is determined by majority voting among the trees. Generally, RF is particularly suitable for effectively managing convoluted datasets and accurately capturing complex correlations among various characteristics. Random Forest is a widely used classification method due to its reduced susceptibility to overfitting compared to standalone decision trees. This characteristic has contributed to its popularity in many classification applications. It is important to acknowledge that the interpretability of RF may differ from that of individual decision trees, mostly owing to the ensemble aspect of the algorithm. See Figure 12.

#### 2.3.8. Deep Learning

**Figure 13:**
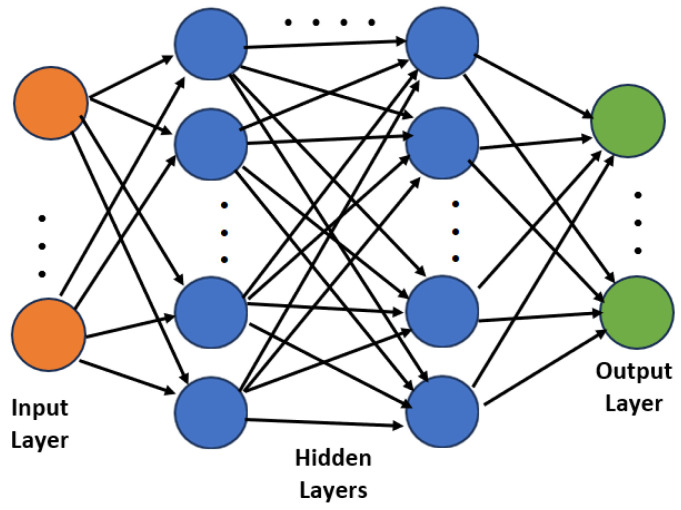
DNN architecture.

The DL network is developed based on the backpropagation algorithm. The neural network consists of multiple layers of interconnected nodes or neurons. It is a feedforward neural network, meaning that information flows in one direction—from the input layer, through the hidden layers, and finally to the output layer. Each connection between nodes has an associated weight, and each node applies an activation function to the weighted sum of its inputs as shown in Figure 13.

The DL model is made up of the following components:

- Input Layer is the first layer in the network, where input data is fed into the model. Each node in the input layer represents a feature of the input data.
- Hidden layers come after the input layer but before the output layer. Each node in a hidden layer performs a weighted sum of its inputs, applies an activation function to the result, and passes the output to the next layer. Multiple hidden layers allow the network to learn complex and hierarchical representations of the input data.
- Output Layer is the final layer that produces the network’s output. The number of nodes in the output layer depends on the type of task the network is designed for. For binary classification, there is typically one node with a sigmoid activation function, while for multi-class classification, there might be multiple nodes with softmax activation.
- Weights and Biases: Each connection between nodes has an associated weight learned during training. Bias refers to a term added to the weighted sum of inputs and passed through an activation function. It allows the neural network to represent constant values in the output, even when all the input values are zero. The weights and biases are adjusted during training to minimize the difference between the predicted output and target values.
- Activation Functions: Nodes in hidden layers and the output layer typically apply an activation function to introduce non-linearity into the model. Common activation functions include the rectified linear unit (ReLU) for hidden layers and the sigmoid as the output layer for binary classification. The ReLU function is computationally efficient and helps mitigate the vanishing gradient problem. It is commonly used in hidden layers of neural networks. Output: [0, +∞) for positive values, 0 for negative values. The sigmoid function squashes its input to the range (0, 1), making it suitable for binary classification problems where the output represents probabilities.
- Stochastic Gradient Descent (SGD) is used for optimization during backpropagation. Instead of updating weights after processing the entire dataset (batch), weights are updated after processing a subset (mini-batch) of the data, which reduces the computational load and helps escape local minima.
- Backpropagation The algorithm compares the predicted output of the network with the actual output (ground truth) and calculates the error. The error is then propagated backward through the network to update weights and reduce errors in subsequent iterations.
- DL training: the network is trained using a supervised learning approach, where it learns from a labeled dataset. The optimization algorithm automatically performs backpropagation during the training process (in this case, the SGD). The algorithm computes the gradient of the loss concerning the weights and biases in the network. This gradient represents the direction in which the weights and biases should be adjusted to decrease the loss. During each training epoch, the model processes batches of training data, computes the loss, and updates its parameters through backpropagation. This iterative process continues for the specified number of epochs, and the model gradually improves its ability to predict the given task.
- Weights are updated using the error gradient with respect to the weights. The learning rate controls the step size during weight updates. After the training progress is completed, the final weights are used for DL prediction.

The built model has the following parameters: 12 inputs; 1 output; learning rate = 0.1; epochs = 100; 2 hidden layers, each containing nine neuron activation functions, which are Relu and sigmoid; batch size = 10. 

### 2.4. Metric Evaluation

To validate the proposed techniques, the following ML factors were calculated: precision, recall, and F1-Score based on the following parameters:

- True Positive (TP): The number of instances that are actually positive (belong to the positive class) and are correctly predicted as positive by the model.
- False Positive (FP): The number of instances that are actually negative (belong to the negative class) but are incorrectly predicted as positive by the model.
- True Negative (TN): The number of instances that are actually negative and are correctly predicted as negative by the model.
- False Negative (FN): The number of instances that are actually positive but are incorrectly predicted as negative by the model.

Precision, also known as positive predictive value, is the ratio of correctly predicted positive instances to the total predicted positive instances. Precision for the negative class is calculated similarly, but it focuses on the instances predicted as negative.

(6) $$\mathrm{Precision}\;\left(\mathrm{Positive}\;\mathrm{class}\right)=\frac{\mathrm{TP}}{\mathrm{TP}+\mathrm{FP}}$$

(7) $$\;\mathrm{Precision}\;\left(\mathrm{Negative}\;\mathrm{Class}\right)=\frac{\mathrm{TN}}{\mathrm{TN}\;+\;\mathrm{FN}}$$

Recall quantifies the number of positive class predictions made from all positive examples in the dataset. Unlike precision, which only comments on the correct positive predictions out of all positive predictions, recall indicates missed positive predictions.

Recall, also known as sensitivity or true positive rate, is the ratio of correctly predicted positive instances to the total actual positive instances.

(8) $$\mathrm{Recall}\;\left(\mathrm{positive}\;\mathrm{class}\right)=\frac{\mathrm{TP}}{\mathrm{TP}+\mathrm{FN}}$$

Recall for the negative class is calculated similarly, but it focuses on the instances predicted as negative:

(9) $$\mathrm{Recall}\;\left(\mathrm{negative}\;\mathrm{class}\right)=\frac{\mathrm{TN}}{\mathrm{TN}+\mathrm{FP}}$$

F1-Score provides a single score that balances the concerns of precision and recall in one number. F-Score delivers a way to combine both precision and recall into a single measure that captures both properties. Once precision and recall have been calculated, the two scores can be combined into the calculation of the F-Measure. As with precision and recall, a poor F-Measure score is 0.0, and a best or perfect F-Measure score is 1.0.

(10) $$F1-\mathrm{Score}\;=\frac{2\times \mathrm{recision}\times \mathrm{Recall}}{\mathrm{Precision}+\mathrm{Recall}}$$

Accuracy is the fraction between the number of correct predictions and the number of overall predictions.

(11) $$\mathrm{Accuracy}\;=\frac{\mathrm{Correct}\;\mathrm{predictions}}{\mathrm{Total}\;\mathrm{predictions}}$$

Weighted average aggregates performance metrics, such as precision, recall, F1 score, etc., by considering the class distribution. The weighted average takes into account the number of instances in each class, providing a more balanced assessment of the overall model performance. It is advantageous when dealing with imbalanced datasets where one class may substantially outnumber the other.

(12) $$\mathrm{Weighted}\;\mathrm{Average}=\;\frac{{\mathrm{Metric}}_{\mathrm{negative}}\times {\;\mathrm{Weight}}_{\mathrm{negative}}+{\;\mathrm{Metric}}_{\mathrm{positive}}\times {\;\mathrm{Weight}}_{\mathrm{positive}}}{\mathrm{Total}\;\mathrm{Samples}}$$

where:

- Metric_negative_ and Metric_positive_ are each class’s performance metrics (e.g., precision, recall, F1 score).
- Weight_negative_ and Weight_positive_ are the number of samples belonging to the negative class and to the positive class, respectively.
- Total Samples is the total number of samples in the dataset.

The macro-average treats each class in the dataset equally, regardless of its size or frequency. It is a simple average of the performance metrics for each class, providing an unweighted measure of the overall model performance.

(13) $$\mathrm{Macro}\;\mathrm{Average}\;=\frac{{\mathrm{Metric}}_{\mathrm{negative}}+{\mathrm{Metric}}_{\mathrm{positive}}}{2}$$

Metric_negative_ Class and Metric_positive_ Class are the performance metrics (for F1 score in this case) for the positive and negative classes, respectively.

## 3. Results and Discussion

### 3.1. Model Comparison and Selection

K-fold cross-validation is a prevalent method in ML and DL that evaluates the effectiveness of a prediction model and reduces the likelihood of overfitting. The process involves splitting the dataset into K subsets or folds and training and evaluating the model K times. A distinct fold is designated as the test set for each iteration, while the remaining K-1 folds are utilized for training. The results are computed by taking the average across K iterations, which ensures a more resilient and dependable performance estimation. In this case, the K-fold cross-validation was carried out with k = 10.

The AI models were trained on the host computer, containing an NVIDIA Quadro P620 with a Pascal GPU with 512 CUDA cores (NVIDIA, Santa Clara, CA, USA). The machine includes 2 GB of GDDR5 memory, an Intel Core i7 vPro-10850H Processor running at 2.70 GHz, and 32 GB of RAM (Intel, Santa Clara, CA, USA).

**Figure 14:**
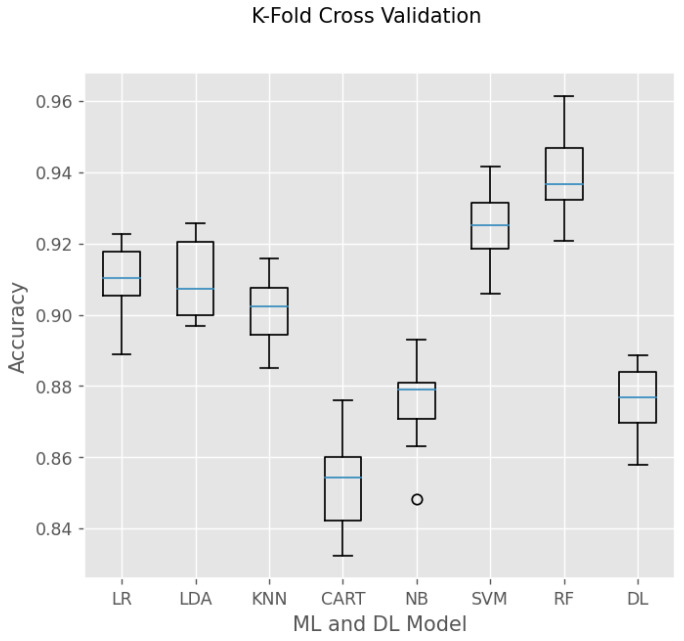
K-fold cross-validation for model evaluations.

As shown in Figure 14 and Table 2, the RF accomplishes the highest accuracy among all the operated models with low standard deviation (std). SVM and RF need more training time, but their accuracy is superior to other ML models. Unlike the RF model, the CART model has the lowest accuracy because the prediction relies on only a single decision tree. Here, RF is constructed by 100 trees, providing more appropriate results. The DL model requires a large training time for a weight update of 100 epochs. Its result is better than the NB and CART models but shows inferior performance compared to LR, LDA, KNN, SVM, and RF. The std of all models is relatively small, which shows these models are trained properly and possess stable performance.

### 3.2. Test on Each Person

In this test, the best algorithm is utilized to test on the data of each person, containing 1440 window samples. After training the ML model, it is tested with 10 different people to validate the system’s accuracy. RF accomplishes the highest accuracy among all algorithms, so it is used as the principal method.

Table 3 reports a good performance of the RF model, which has an accuracy always ≥90%. Furthermore, the precision, recall, and F1-score are equivalently balanced. Only person 3 has a difference between no beating (negative) and heart beating (positive) in the recall and precision metrics that is more relevant. 

With a precision of 0.99 for the positive class, out of all instances predicted as positive by the model, 99% are actually true positive instances. With a precision of 0.91 for the negative class, it means that out of all instances predicted as negative by the model, 91% are actually true negative instances.

With a recall of 0.82 for the positive class, the model correctly identified and classified 82% of the actual positive instances. A recall value of 0.99 for the negative class indicates that the model is highly effective in identifying and capturing 99% of the actual negative instances. This metric suggests a low rate of false negatives in the negative class.

Macro average and weighted average of the F1-score indicate high overall performance across the two classes. The macro average considers both classes equally. In a binary classification scenario, this means that the average performance for both the positive and negative classes is determined. This metric suggests that the model is achieving a high level of correctness and completeness in its predictions across both classes.

The weighted average takes into account the class imbalance by considering the proportion of samples in each class. The weighted average ≥ 0.9, it means that the model is performing well, and the performance is not skewed by the class distribution. Both the positive and negative classes contribute proportionally to the overall performance.

Overall, the model makes accurate and reliable predictions for both positive and negative instances to detect the heart pulse effectively. The mean accuracy of 10 people prediction is 0.93, which is high reliability in ML performance.

## 4. Conclusions

The theme of continuous monitoring of physiological parameters is crucial in the field of Digital Prevention. Wearable devices, particularly smartwatches, serve as the main monitoring tools used nowadays. However, this approach still has problems and limitations in terms of comfort and usability. For this reason, investigating non-invasive monitoring solutions that do not use wearable devices is essential for DP.

In this work, several ML algorithms were analyzed and compared in the task of heartbeat monitoring by a bed-mounted MEMS accelerometer signals. Ten volunteers participated in a lab measurement campaign, producing 2 h of accelerometer signal traces. The 10 users lay on the bed in 4 different positions—in each position, they generated 3 min of recording, for a total of 120 min. In addition to the MEMS sensor, a photoplethysmography sensor was used to establish ground truth. These data were then used to train and test the identified ML models. The ML models were compared to each other to validate their accuracy and the reliability of the experimental setup—the results obtained were decidedly positive, especially in the case of the RF (Random Forest) model (mean accuracy prediction is approximately 0.93).

Out of all the models that were examined, the Random Forest algorithm stands out for having the best level of accuracy. In addition, the research further investigates Deep Learning, especially utilizing backpropagation to train neural networks. The training time is slower than models such as NB and LDA, but notably quicker than SVM and DL models. The findings provide useful insights into the capabilities and constraints of both ML and DL algorithms, enabling guidance for creating heart rate monitoring systems.

This work supports the development of non-invasive techniques for heart rate monitoring without the aid of wearable devices. The developed system, useful mainly during the night, allows the user to monitor their heart rate simply by lying in bed, without the need to wear sensors and avoiding the burden of charging the device. With respect to other approaches (based for instance on smart pads), the adoption of a tiny, solid-state accelerometric sensor allows for a more practical, inexpensive, and less constrained placement of the sensing equipment, making it suitable for effective deployment in real-world care environments.

The present discussion aims at proof of concept only, so that signal processing was carried out offline: an embedded microcontroller system (IoT Discovery Kit) has been used only to acquire the signals and send them to a PC via a fast serial connection, while the developed ML algorithms have been implemented in Python language. Nevertheless, the validated approaches lend themselves to the direct implementation in the same embedded system platform, thus accounting for real-time analysis: using the Wi-Fi connectivity, the results of the analyses will be sent to a cloud infrastructure, to obtain a smart bed compliant with the IoT paradigm.

## Figures and Tables

**Table 1:** ML feature and classification.

ML Features												Heart Beat Class
Xsum (0)	Xstd (1)	Xmax (2)	ΔXsum (3)	ΔXstd (4)	ΔXmax (5)	Ysum (6)	Ystd (7)	Ymax (8)	Zsum (9)	Zstd (10)	Zmax (11)	0 or 1

**Table 2:** AI models comparison.

Models	Mean Accuracy	Std	Training and Test Time for 1-Fold (s)
LR	0.908	0.09	0.179
LDA	0.902	0.013	0.048
KNN	0.907	0.011	0.069
CART	0.854	0.015	0.2385
NB	0.846	0.012	0.006
SVM	0.925	0.014	3.246
RF	0.935	0.012	2.256
DL	0.876	0.010	109.105

**Table 3:** RF performance for each person in test.

Person Index	Random Forest								
	Precision		Recall		F1-Score		Macro Average	Weighted Average	Accuracy
	No Beating	Heart Beating	No Beating	Heart Beating	No Beating	Heart Beating			
P1	0.90	0.95	0.95	0.89	0.93	0.92	0.92	0.92	0.92
P2	0.91	0.94	0.89	0.95	0.90	0.95	0.92	0.93	0.93
P3	0.91	0.99	0.99	0.82	0.95	0.89	0.92	0.93	0.93
P4	0.91	0.93	0.93	0.90	0.92	0.92	0.92	0.92	0.92
P5	0.96	0.95	0.96	0.95	0.96	0.95	0.96	0.96	0.96
P6	0.93	0.93	0.94	0.92	0.94	0.92	0.95	0.93	0.93
P7	0.88	0.91	0.86	0.93	0.87	0.92	0.90	0.90	0.90
P8	0.96	0.93	0.94	0.96	0.95	0.95	0.95	0.95	0.95
P9	0.87	0.96	0.95	0.90	0.91	0.93	0.91	0.92	0.92
P10	0.93	0.93	0.90	0.95	0.92	0.94	0.93	0.93	0.93

## Data Availability

Data are contained within the article.
